# Traditional Chinese medicine use is associated with lower risk of pneumonia in patients with systemic lupus erythematosus: a population-based retrospective cohort study

**DOI:** 10.3389/fphar.2023.1185809

**Published:** 2023-06-01

**Authors:** Weijie Wang, Yu-Hsun Wang, Kepeng Yang, Xiangsheng Ye, Xinchang Wang, James Cheng-Chung Wei

**Affiliations:** ^1^ Department of Rheumatology, The Second Affiliated Hospital of Zhejiang Chinese Medical University, Hangzhou, China; ^2^ Institute of Basic Theory for Chinese Medicine, China Academy of Chinese Medical Science, Beijing, China; ^3^ Department of Medical Research, Chung Shan Medical University Hospital, Taichung, Taiwan; ^4^ The First Clinical Medical College, Zhejiang Chinese Medical University, Hangzhou, China; ^5^ Department of Allergy, Immunology and Rheumatology, Chung Shan Medical University Hospital, Taichung, Taiwan; ^6^ Institute of Medicine, Chung Shan Medical University, Taichung, Taiwan; ^7^ Graduate Institute of Integrated Medicine, China Medical University, Taichung, Taiwan; ^8^ Department of Medical Research, Taichung Veterans General Hospital, Taichung, Taiwan

**Keywords:** systemic lupus erythematosus (SLE), traditional Chinese medicine (TCM), pneumonia, cohort, infection

## Abstract

**Objectives:** To investigate the association between traditional Chinese medicine (TCM) therapy and the risk of pneumonia in patients with systemic lupus erythematosus (SLE).

**Methods:** This population-based control study analyzed the data retrieved from the National Health Insurance Research database in Taiwan. From a cohort of 2 million records of the 2000–2018 period, 9,714 newly diagnosed patients with SLE were initially included. 532 patients with pneumonia and 532 patients without pneumonia were matched 1:1 based on age, sex, and year of SLE diagnosis using propensity score matching. The use of TCM therapy was considered from the SLE diagnosis date to the index date and the cumulative days of TCM therapy were used to calculate the dose effect. Conditional logistic regression was used to investigate the risk of pneumonia infection. Furthermore, to explore the severity of pneumonia in SLE, sensitivity analyses were performed after stratification using the parameters of emergency room visit, admission time, and antibiotic use.

**Results:** TCM therapy for >60 days could significantly reduce the risk of pneumonia in patients with SLE (95% CI = 0.46–0.91; *p* = 0.012). Stratified analysis showed that TCM use also reduced the risk of pneumonia in younger and female patients with SLE by 34% and 35%, respectively. TCM for >60 days significantly reduced the risk of pneumonia in the follow-up periods of >2, >3, >7, and >8 years. In addition, the exposure of TCM for >60 days reduced the risk of pneumonia in patients with SLE who were treated with antibiotics for moderate or severe pneumonia. Finally, the study found that using formulae to tonify the kidney for more than 90 days and formulae to activate blood circulation for less than 30 days could significantly reduce the risk of pneumonia infection in patients with SLE.

**Conclusion:** TCM use is associated with a lower risk of pneumonia among patients with SLE.

## Introduction

Systemic lupus erythematosus (SLE) is an autoimmune disease that presents with complex clinical manifestations. SLE can affect all systems of the body, including the kidneys and nervous system (Kiriakidou and Ching, 2020). The estimated worldwide prevalence of SLE is approximately 30–50/100,000 people, and the prevalence is higher in developing countries (Ingvarsson et al., 2016; Tsioni et al., 2015; Li et al., 2012). The 10-year survival rate is approximately 90%, 15-year survival rate is 85%, and 20-year survival rate is 78% (Durcan et al., 2019), indicating that the disease seriously affects the physical and mental health of the patients as well as their quality of life. The current management of SLE is still dominated by glucocorticoids and immunosuppressive agents. However, these agents often cause several side effects, such as a secondary infection (27%), hypertension (11.3%), and osteoporosis (7.5%) (Tektonidou et al., 2015). Among the side effects, infection and lupus nephritis are the main causes of death in SLE (Sciascia et al., 2017). Clinical studies have found that belimumab, the first biologic approved for the treatment of SLE by the Food and Drug Administration, combined with standard therapy can alleviate the disease activity in some patients with SLE and has been shown to have a safety profile similar to a placebo (Stohl et al., 2017). Although pre-emptive antimicrobial therapy and vaccination has become the consensus for preventing infections, the incidence of certain infections, such as pneumonia, remains high and inevitable (Oku et al., 2021). Therefore, there is an urgent need for the development of clinical treatments or drugs with high safety and good efficacy for alleviating SLE and preventing infections.

Traditional Chinese medicine (TCM) has been used for treating several autoimmune diseases (called Bi syndrome in TCM), including SLE, and has shown significant clinical efficacy for thousands of years (Wang et al., 2019). We previously conducted a meta-analysis of 13 randomized, placebo-controlled trials that included 856 participants and revealed that TCM could control disease activity and reduce glucocorticoid dose used among patients with SLE (Wang et al., 2021). A population-based cohort study based on the National Health Insurance Research Database (NHIRD) in Taiwan provided evidence that regular treatment combined with Chinese herbal medicine improves the survival of patients with SLE. The study included several useful TCM formulae, including Zhi Bo Di Huang Wan, Jia Wei Xiao Yao San, Liu Wei Di Huang Wan, Gan Lu Yin, and Yin Qiao San (Ma et al., 2016). However, few studies have evaluated whether TCM can reduce the risk of infections in patients with SLE.

To the best of our knowledge, no large-scale study has evaluated the association between TCM therapy and the risk of pneumonia in patients with SLE. Thus, the present study aimed to provide some evidence regarding the aforementioned association.

## Methods

### Data sources

The research data of 2 million people from 1 January 2000, to 31 December 2018, were retrieved from NHIRD, Taiwan, out of the 23 million people included in the database. The random sampling method was employed, with serial numbers assigned to each of the 23 million insurance beneficiaries. NHIRD contains information on patient demographics, age, sex, disease diagnoses, number of clinical visits and hospitalizations, prescribed medications (with dosages), and TCM treatment agents administered by registered TCM physicians. Diseases were defined as per the International Classification of Diseases (ICD) Ninth Revision (ICD-9) and 10th Edition (ICD-10) codes (Goulielmos and Zervou, 2020).

### Patients

**FIGURE 1:**
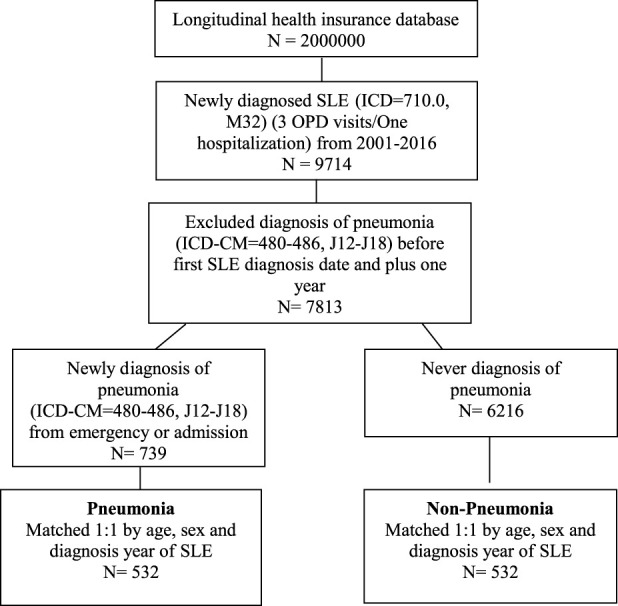
Flow chart of the study design. SLE, systemic lupus erythematosus; OPD, Outpatient department.

As shown in Figure 1 total of 2 million patients were randomly selected from NHIRD. The included patients were those who were newly diagnosed with SLE (ICD-9 code = 710.0; ICD-10 code = M32) from January 2001 to December 2016 (*n* = 9,714). A stringent criterion of requiring at least three outpatient visits or one hospital admission was used for recognizing SLE. In addition, pneumonia (ICD-9 code = 480–486; ICD-10 code = J12–J18) was chosen as the representative infection with SLE. After excluding the diagnosis of pneumonia before the first SLE diagnosis date and 1 year after SLE diagnosis, a total of 7,813 patients with SLE were finally selected as the study group. A total of 739 pneumonia patients with SLE were selected from the emergency or admission group. The index date was defined as the pneumonia onset date (Supplementary Figure S1). Moreover, 6,216 patients with SLE were never diagnosed with pneumonia. After 1:1 propensity score matching using the parameters of age, sex, and SLE diagnosis year, 532 pneumonia and 532 non-pneumonia patients with SLE were included in the analysis.

### Traditional Chinese medicine and covariates

TCM use was calculated from the SLE diagnosis date to the index date, and the cumulative days of TCM therapy were used to calculate the dose effect. TCM users were defined as patients who used TCM more than three times. In addition, the cumulative days of TCM therapy were classified as 30, 60, and 90 days. TCM considers SLE as a systemic disease associated with the state of the entire body and implicates toxic heat, blood stasis, and kidney yin deficiency in the pathogenesis of SLE (Sun et al., 2018). Therefore, the TCM formulae were initially classified into three types: those tonifying the kidney (KF), those activating blood circulation (BF), and the “other TCM therapy” group. The classification standards were followed as per the classification criteria described in a previous study (Lin et al., 2021). As a result, the top 20 frequently used Chinese medicine formulae in NHIRD were selected and classified into two groups: the KF and BF groups. The KF group included Guilu Erxian Jiao, Jisheng Shenqi Pill, Qiju Dihuang Pill, Zhibo Dihuang Pill, Liuwei Dihuang Wan, and Zuo Guiwan, whereas the BF group comprised Duhuo Jisheng Decoction, Shujing Huoxue Decoction, Danggui Niantong Decoction, Shaoyao Gancao Decoction, Shentong Zhuyu Decoction, Xuefu Zhuyu Decoction, Xiao Huoluo Dan, and Guizhi Shaoyao Zhimu Decoction (Supplementary Table S1). The baseline comorbidities included hypertension (ICD-9 code = 401–405; ICD-10 code = I10–I15), hyperlipidemia (ICD-9 code = 272; ICD-10 code = E78), chronic liver disease (ICD-9 code = 571; ICD-10 code = K70, K73, K74, K75.4, K75.81, K76.0, K76.89, and K76.9), chronic kidney disease (ICD-9 code = 585; ICD-10 code = N184, N185, N186, and N189), diabetes (ICD-9 code = 250; ICD-10 code = E10, E11, E12, E13, and E14), chronic obstructive pulmonary disease (ICD-9 code = 491, 492, and 496; ICD-10 code = J41–J44), rheumatoid arthritis (ICD-9 code = 714.0; ICD-10 code = M05 and M06), ankylosing spondylitis (ICD-9 code = 720.0; ICD-10 code = M45 and M46), hepatitis B (ICD-9 code = 070.2, 070.3, and V02.61; ICD-10 code = B16.0, B16.1, B16.2, B16.9, B18.0, B18.1, B19.10, B19.11, and Z22.51), hepatitis C (ICD-9 code = 070.41, 070.44, 070.51, 070.54, 070.7, and V02.62; ICD-10 code = B17.10, B17.11, B18.2, B19.20, B19.21, and Z22.52), endocarditis (ICD-9 code = 424.91; ICD-10 code = I39), nephritis (ICD-9 code = 583.81; ICD-10 code = E10.21, E11.21, and N16), glomerulonephritis (ICD-9 code = 580.81, 581.81, and 582.81; ICD-10 code = N08). These comorbidities were identified during at least three outpatient visits or one hospital admission between the SLE diagnosis date and index date. The Anatomical Therapeutic Chemical (ATC) Classification System codes were used for defining drugs. The use of corticosteroids, hydroxychloroquine (ATC code = P01BA02), and methotrexate (ATC code = L04AX03) for at least three times prescription in each kind of medication between the SLE diagnosis date and index date was evaluated.

### Statistical analysis

The Chi-squared test and Student’s *t*-test were used to compare continuous and dichotomous data, respectively, between the pneumonia and non-pneumonia groups. Multivariate conditional logistic regression analysis was performed to evaluate the risk of pneumonia infection in patients with SLE after prescribing TCM therapy. The risk was estimated using adjusted odds ratios (aORs) and 95% confidence intervals (CIs). Statistical significance was defined as *p-*value <0.05, and all data analyses were performed using the statistical package SAS for Windows (Version 9.4, SAS Institute Inc., Carey, NC, United States).

Subgroup analyses were performed to determine how the risk of pneumonia infection in patients with SLE patients differed as per sex, age, and days of TCM use. In addition, the association between the use of different TCM formulae and risk of pneumonia infection was evaluated. Antibiotic use and hospitalization period were analyzed to estimate the severity of pneumonia.

## Results

### Demographic characteristics and comorbidities

The demographic characteristics of included patients with SLE in the pneumonia and non-pneumonia groups are shown in Table 1. After 1:1 propensity score matching using the parameters of age, sex, and SLE diagnosis year, 532 pneumonia and 532 non-pneumonia patients with SLE were finally included in the study. SLE patients with pneumonia had several comorbidities, including hypertension (*p* = 0.0014), chronic liver disease (*p* = 0.0312), chronic kidney disease (*p* < 0.001), diabetes (*p* = 0.0002), chronic obstructive pulmonary disease (COPD) (*p* = 0.0102), and rheumatoid arthritis (*p* = 0.0308), except ankylosing spondylitis. Moreover, corticosteroids, nonsteroidal anti-inflammatory drugs, and hydroxychloroquine were used more frequently by patients with SLE in the pneumonia group. Finally, TCM was prescribed more frequently to SLE patients with pneumonia in the 60 days stratification groups (*p* = 0.0268).

**TABLE 1:** Demographic characteristics of pneumonia and non-pneumonia.

	Non-pneumonia (*N* = 532)	Pneumonia (*N* = 532)	*p*-value
Age			1
<65	413 (77.6)	413 (77.6)	
≥65	119 (22.4)	119 (22.4)	
Mean ± SD	50.6 ± 17	50.6 ± 17	1
Age			1
<50	245 (46.1)	245 (46.1)	
≥50	287 (53.9)	287 (53.9)	
Sex			1
Female	452 (85.0)	452 (85.0)	
Male	80 (15.0)	80 (15.0)	
TCM	295 (55.5)	262 (49.2)	0.0428
TCM (day)			0.0640
None	237 (44.5)	270 (50.8)	
≤30	107 (20.1)	108 (20.3)	
31–60	42 (7.9)	44 (8.3)	
>60	146 (27.4)	110 (20.7)	
TCM (days)			0.0117
None	237 (44.5)	270 (50.8)	
≤30	107 (20.1)	108 (20.3)	
31–60	42 (7.9)	44 (8.3)	
61–90	31 (5.8)	11 (2.1)	
>90	115 (21.6)	99 (18.6)	
TCM (days)			0.1233
None	237 (44.5)	270 (50.8)	
≤90	180 (33.8)	163 (30.6)	
>90	115 (21.6)	99 (18.6)	
TCM (days)			0.0629
None	237 (44.5)	270 (50.8)	
≤30	107 (20.1)	108 (20.3)	
>30	188 (35.3)	154 (28.9)	
TCM (days)			0.0268
None	237 (44.5)	270 (50.8)	
≤60	149 (28.0)	152 (28.6)	
>60	146 (27.4)	110 (20.7)	
Hypertension	149 (28.0)	198 (37.2)	0.0014
Hyperlipidemia	117 (22.0)	140 (26.3)	0.0995
Chronic liver disease	74 (13.9)	100 (18.8)	0.0312
Chronic kidney disease	25 (4.7)	61 (11.5)	<0.001
Diabetes	52 (9.8)	94 (17.7)	0.0002
Chronic obstructive pulmonary disease	40 (7.5)	65 (12.2)	0.0102
Rheumatoid arthritis	53 (10.0)	76 (14.3)	0.0308
Ankylosing spondylitis	15 (2.8)	4 (0.8)	0.0109
Corticosteroids	503 (94.5)	522 (98.1)	0.0019
NSAIDs	512 (96.2)	523 (98.3)	0.0384
Hydroxychloroquine	141 (26.5)	234 (44.0)	<0.001
Methotrexate	33 (6.2)	50 (9.4)	0.0520
Follow-up duration (years)	7.1 ± 4.4	7.1 ± 4.4	0.9358
SLE year			1.0
2001	96 (18.0)	96 (18.0)	
2002	66 (12.4)	66 (12.4)	
2003	61 (11.5)	61 (11.5)	
2004	50 (9.4)	50 (9.4)	
2005	47 (8.8)	47 (8.8)	
2006	38 (7.1)	38 (7.1)	
2007	30 (5.6)	30 (5.6)	
2008	24 (4.5)	24 (4.5)	
2009	22 (4.1)	22 (4.1)	
2010	25 (4.7)	25 (4.7)	
2011	16 (3.0)	16 (3.0)	
2012	21 (3.9)	21 (3.9)	
2013	13 (2.4)	13 (2.4)	
2014	7 (1.3)	7 (1.3)	
2015	10 (1.9)	10 (1.9)	
2016	6 (1.1)	6 (1.1)	

NSAIDs, Non-steroidal anti-inflammatory drugs; SLE, systemic lupus erythematosus.

### TCM use reduced the risk of pneumonia in SLE patients

Conditional logistic regression analysis revealed that TCM use reduced the risk of pneumonia in patients with SLE (aOR (95% CI) = 0.73 (0.55–0.96); *p* = 0.026). Among the comorbidities, chronic kidney disease (aOR (95% CI) = 2.23 (1.25–3.97); *p* = 0.007), diabetes (aOR (95% CI) = 1.84 (1.18–2.87); *p* = 0.007), and COPD (aOR (95% CI) = 1.72 (1.06–2.79); *p* = 0.028) were associated with a higher risk of pneumonia in patients with SLE. Moreover, the use of corticosteroids (aOR (95% CI) = 2.99 (1.31–6.83); *p* = 0.009) and hydroxychloroquine (aOR (95% CI) = 2.02 (1.48–2.74); *p* < 0.001) increased the risk of pneumonia in patients with SLE (Table 2).

**TABLE 2:** Conditional logistic regression of risk of pneumonia.

	cOR (95% C.I.)	*p*-value	aOR^a^ (95% C.I.)	*p*-value
TCM	0.73 (0.55–0.96)	0.026	0.73 (0.55–0.96)	**0.026**
Hypertension	1.78 (1.31–2.421)	<0.001	1.18 (0.81–1.71)	0.397
Hyperlipidemia	1.31 (0.97–1.77)	0.080	1.20 (0.83–1.74)	0.331
Chronic liver disease	1.47 (1.05–2.07)	0.027	1.17 (0.80–1.71)	0.423
Chronic kidney disease	2.80 (1.68–4.67)	<0.001	2.23 (1.25–3.97)	**0.007**
Diabetes	2.17 (1.46–3.22)	<0.001	1.84 (1.18–2.87)	**0.007**
Chronic obstructive pulmonary disease	1.81 (1.17–2.80)	0.008	1.72 (1.06–2.79)	**0.028**
Rheumatoid arthritis	1.52 (1.04–2.23)	0.030	1.33 (0.85–2.10)	0.211
Ankylosing spondylitis	0.27 (0.09–0.80)	0.019	0.34 (0.10–1.08)	0.067
Corticosteroids	3.11 (1.47–6.59)	0.003	2.99 (1.31–6.83)	**0.009**
NSAIDs	2.38 (1.04–5.43)	0.040	1.88 (0.72–4.92)	0.201
Hydroxychloroquine	2.26 (1.72–2.97)	<0.001	2.02 (1.48–2.74)	**<0.001**
Methotrexate	1.59 (1.00–2.53)	0.052	1.04 (0.60–1.78)	0.897

Bold font indicates statistical significance (*p* < 0.05). cOR, crude odds ratio; aOR, adjusted odds ratio; COPD, chronic obstructive pulmonary disease; NSAIDs, Non-steroidal anti-inflammatory drugs.

^a^ Adjusted for all variables.

## Subgroup analyses

### Association between TCM use and risk of pneumonia at different sub-doses

The number of days of TCM use was considered as per our previous research (Liang et al., 2021; Lin et al., 2021). Thus, 30 days was selected as an interval, and 30, 60, and 90 days were selected to estimate the risk. When 60 days of TCM use was used for stratification, the statistical difference between the two groups was evident (Table 1). Thus, patients with SLE who cumulatively received TCM for >60 days had a significantly lower risk of pneumonia (aOR (95% CI) = 0.64 (0.45–0.90); *p* = 0.011) (Table 3).

**TABLE 3:** Conditional logistic regression of risk of pneumonia in different sub-doses (60 days).

	cOR (95% C.I.)	*p*-value	aOR^a^ (95% C.I.)	*p*-value
TCM (day)				
None	Reference		Reference	
≤60	0.89 (0.67–1.18)	0.405	0.81 (0.59–1.12)	0.205
>60	0.66 (0.49–0.90)	0.008	0.64 (0.45–0.90)	0.011
Hypertension	1.78 (1.31–2.421)	<0.001	1.19 (0.82–1.73)	0.361
Hyperlipidemia	1.31 (0.97–1.77)	0.080	1.22 (0.84–1.76)	0.302
Chronic liver disease	1.47 (1.05–2.07)	0.027	1.18 (0.81–1.73)	0.399
Chronic kidney disease	2.80 (1.68–4.67)	<0.001	2.22 (1.24–3.95)	0.007
Diabetes	2.17 (1.46–3.22)	<0.001	1.81 (1.16–2.82)	0.009
Chronic obstructive pulmonary disease	1.81 (1.17–2.80)	0.008	1.72 (1.06–2.79)	0.028
Rheumatoid arthritis	1.52 (1.04–2.23)	0.030	1.31 (0.83–2.06)	0.250
Ankylosing spondylitis	0.27 (0.09–0.80)	0.019	0.35 (0.11–1.13)	0.080
Corticosteroids	3.11 (1.47–6.59)	0.003	2.97 (1.30–6.77)	0.010
NSAIDs	2.38 (1.04–5.43)	0.040	1.85 (0.70–4.88)	0.212
Hydroxychloroquine	2.26 (1.72–2.97)	<0.001	2.00 (1.47–2.72)	<0.001
Methotrexate	1.59 (1.00–2.53)	0.052	1.08 (0.62–1.85)	0.794

cOR, crude odds ratio; aOR, adjusted odds ratio; NSAIDs, Non-steroidal anti-inflammatory drugs.

^a^ Adjusted for all variables.

### Association between TCM use and risk of pneumonia in different sub populations and follow-up periods

**FIGURE 2:**
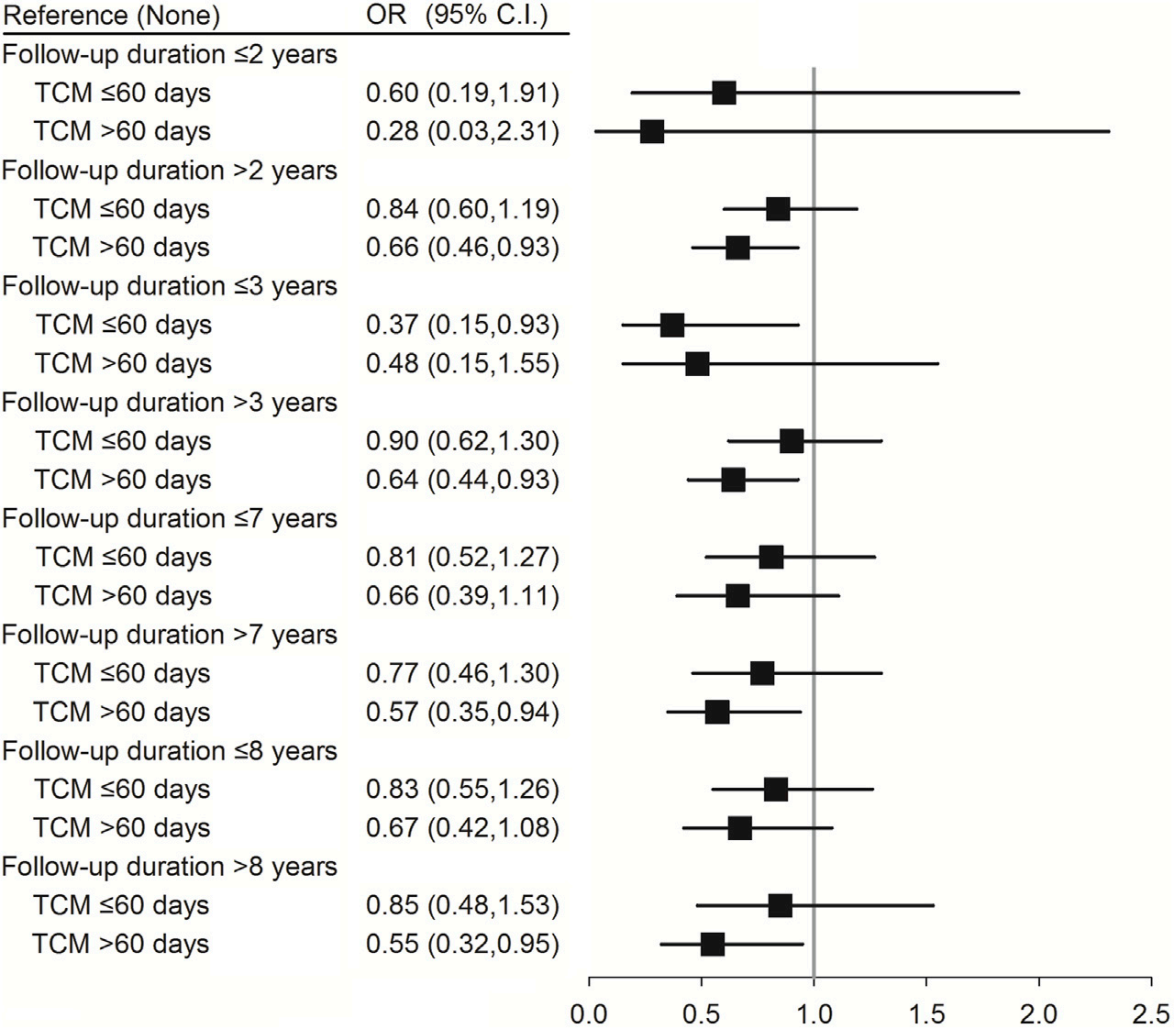
Conditional logistic regression of risk of pneumonia by follow-up duration stratification (60 days). †Adjusted for hypertension, hyperlipidemia, chronic liver disease, chronic kidney disease, rheumatoid arthritis, corticosteroids, NSAIDs, and hydroxychloroquine.

Conditional logistic regression analysis was performed to evaluate the association between TCM use and the risk of pneumonia in patients with SLE according to age, sex, and follow-up duration, which was defined from the SLE diagnosis date to index date. As shown in Table 4, using TCM for >60 days reduced the risk of pneumonia in younger patients with SLE (age <65 years) (aOR (95% CI) = 0.66 (0.44–0.99); *p* = 0.042). In addition, female patients with SLE had a lower risk of pneumonia after using TCM for >60 days (aOR (95% CI) = 0.65 (0.45–0.93); *p* = 0.019). Moreover, patients who used TCM for >60 days and followed-up for >2 (aOR (95% CI) = 0.66 (0.46–0.93); *p* = 0.034), >3 (aOR (95% CI) = 0.64 (0.44–0.93); *p* = 0.019), >7 (aOR (95% CI) = 0.57 (0.35–0.94); *p* = 0.028), and >8 years (aOR (95% CI) = 0.55 (0.32–0.95); *p* = 0.032) had a lower risk of pneumonia (Figure 2; Supplementary Table S2).

**TABLE 4:** Conditional logistic regression of risk of pneumonia by age, sex stratification (60 days).

	N	No. of pneumonia	aOR (95% C.I.)	*p*-value
Age <65				
TCM (day)				
None	392	209	References	
≤60	228	112	0.77 (0.53–1.12)	0.167
>60	206	92	0.66 (0.44–0.99)	0.042
Age ≥ 65				
TCM (day)				
None	115	61	References	
≤60	73	40	1.04 (0.52–2.08)	0.920
>60	50	18	0.52 (0.25–1.05)	0.069
Female				
TCM (day)				
None	408	220	References	
≤60	264	134	0.85 (0.60–1.20)	0.344
>60	232	98	0.65 (0.45–0.93)	0.019
Male				
TCM (day)				
None	99	50	References	
≤60	37	18	0.58 (0.21–1.56)	0.277
>60	24	12	0.59 (0.19–1.77)	0.343

^a^ Adjusted for hypertension, hyperlipidemia, chronic liver disease, chronic kidney disease, diabetes, chronic obstructive pulmonary disease, rheumatoid arthritis, ankylosing spondylitis, corticosteroids, NSAIDs, hydroxychloroquine, and methotrexate.

## Sensitivity analyses

### Association between TCM use and risk of pneumonia as per antibiotic use, severity of pneumonia, and days of hospitalization

**FIGURE 3:**
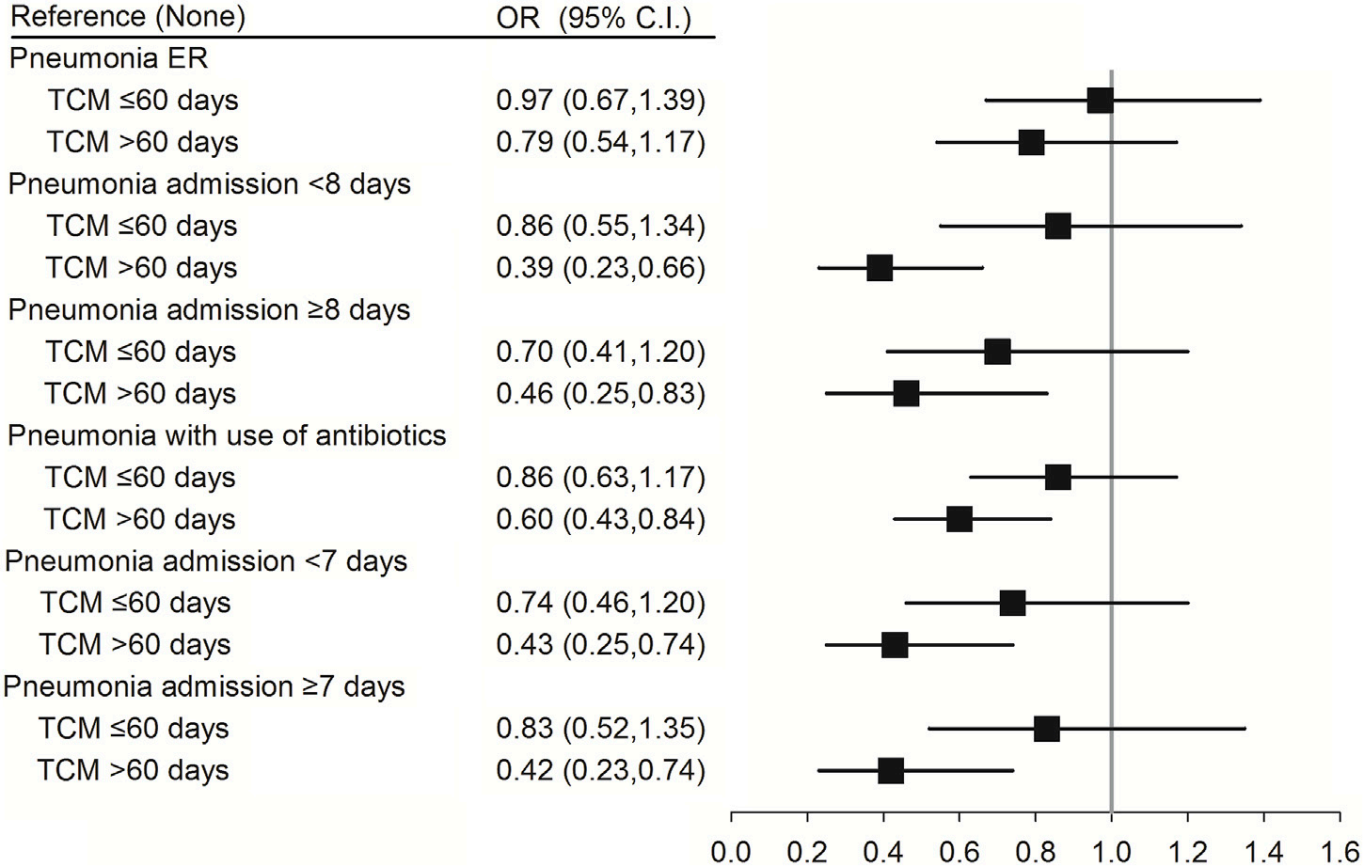
Conditional logistic regression of risk of pneumonia by antibiotics use, severity of pneumonia and days of hospitalization. ER, emergency room. †Adjusted for all variables.

Antibiotic use and days of hospitalization may vary as per the severity of pneumonia in patients with SLE. Compared with patients with SLE in the non-pneumonia group, TCM use for >60 days reduced the risk of pneumonia with antibiotic use (aOR (95% CI) = 0.60 (0.43–0.84); *p* = 0.005) in pneumonia group. Moreover, TCM use for >60 days reduced the risk of pneumonia in patients with SLE having moderate (pneumonia admission <7 days) (aOR (95% CI) = 0.43 (0.25–0.74); *p* = 0.002) or severe pneumonia [(pneumonia admission ≥7 days) (aOR (95% CI) = 0.42 (0.23–0.74); *p* = 0.003) and (pneumonia admission ≥8 days) (aOR (95% CI) = 0.46 (0.25–0.83); *p* = 0.010)]. However, there were no significant differences between pneumonia patients from the emergency room and non-pneumonia patients (*p* = 0.859 or *p* = 0.241) (Figure 3; Supplementary Table S3).

### Association between the use of different TCM formulae and risk of pneumonia

**FIGURE 4:**
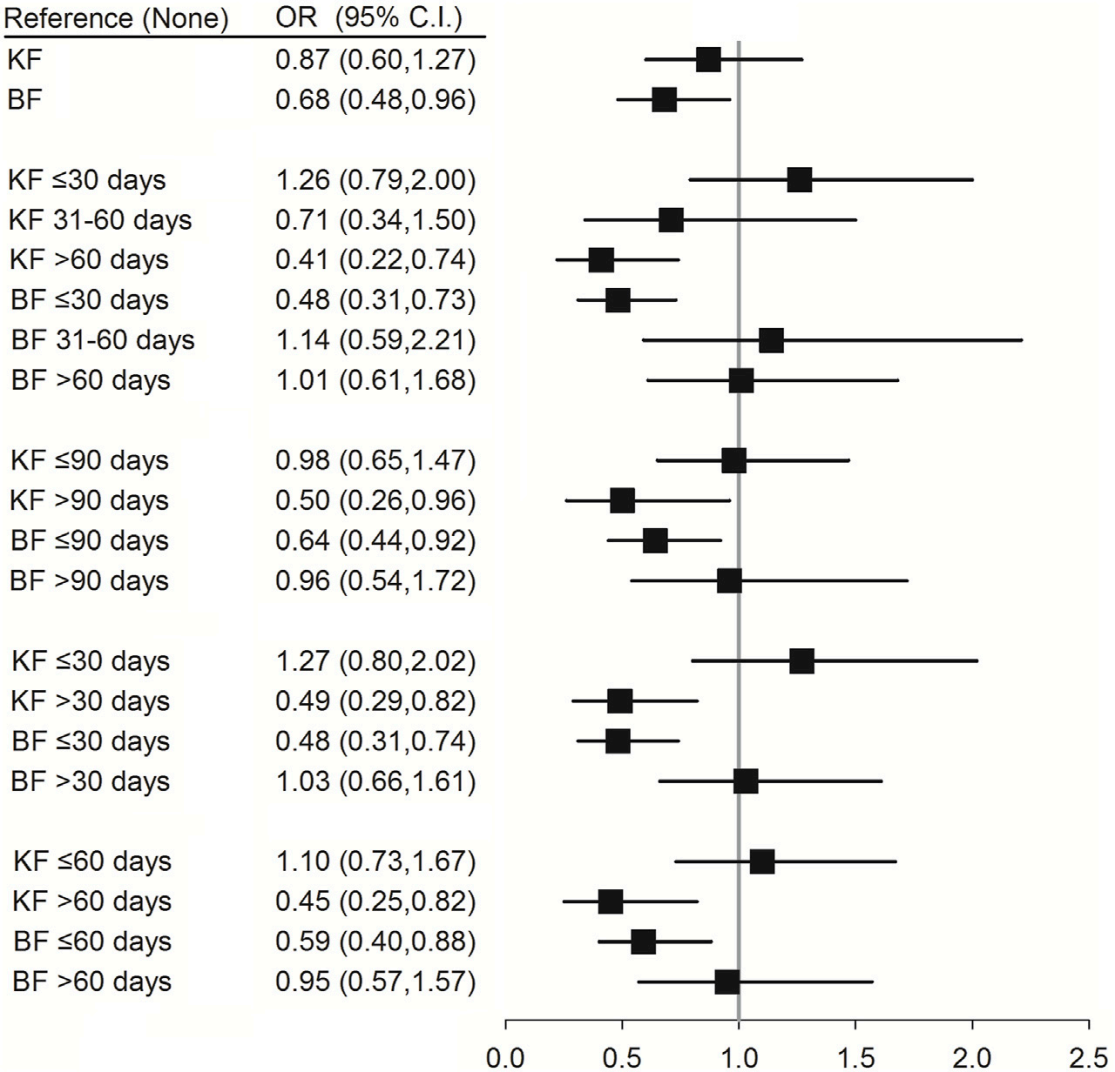
Conditional logistic regression of risk of pneumonia by different formulae of TCM. † Adjusted for hypertension, hyperlipidemia, chronic liver disease, chronic kidney disease, diabetes, chronic obstructive pulmonary disease, rheumatoid arthritis, ankylosing spondylitis, corticosteroids, NSAIDs, hydroxychloroquine, and methotrexate. KF: Chinese formulae tonifying the kidney; BF: Chinese formulae activating blood circulation.

Conditional logistic regression analysis was performed to evaluate the association between the use of different TCM formulae and risk of pneumonia infection. The results showed that patients with SLE who used the formulae of the KF group for >30 days (OR (95% CI) = 0.49 (0.29–0.82); *p* = 0.007), >60 days (OR (95% CI) = 0.45 (0.25–0.82); *p* = 0.008), and >90 days (OR (95% CI) = 0.50 (0.26–0.96); *p* = 0.038) had a reduced risk of pneumonia. Moreover, patients with SLE who used the formulae of the BF group for ≤30 days had a reduced risk of pneumonia (OR (95% CI) = 0.48 (0.31–0.74); *p* < 0.001) (Figure 4; Supplementary Table S4).

## Discussion

To the best of our knowledge, this is the first large-scale cohort study to demonstrate that TCM decreases the risk of pneumonia in patients with SLE. The study results revealed that patients with SLE who cummulatively used TCM for >60 days had a significantly lower risk of pneumonia. A 34% and 35% reduced risk of pneumonia, respectively, was observed in younger and female patients with SLE. TCM use for >60 days significantly reduced the risk of pneumonia in the follow-up periods of >2, >3, >7, and >8 years. In addition, TCM use for >60 days reduced the risk of pneumonia in patients with SLE who also used antibiotics for moderate or severe pneumonia. Furthermore, the use of formulae of the KF group for >90 days and use of formulae of the BF group for <30 days significantly reduced the risk of pneumonia infection in patients with SLE.

SLE is a chronic autoimmune disease with multiorgan manifestations. On the one hand, infection is a common triggering factor that activates SLE and also a common cause of mortality in SLE. The Epstein–Barr virus (Truszewska et al., 2021)and some bacterial lipopolysaccharides (Jung and Suh, 2017) have been reported as pivotal factors for inducing SLE. Bonometti et al. illustrated the case of a patient in whom SLE was triggered by coronavirus disease 2019 infection (Bonometti et al., 2020). On the other hand, a secondary infection has become a common cause of mortality (25%–50%) in SLE (Wang et al., 2015; Kedves et al., 2020). A previous study revealed that lung, cutaneous, and urinary tract infections account for more than two-thirds of all infections in SLE (Jeong et al., 2009). The risk factors of infection in patients with SLE include the overall disease activity, higher C-reactive protein levels, higher anti-dsDNA levels, low complement levels, nephritis, daily dose of prednisone>10 mg, and others (Duffy et al., 1991; Suh et al., 2001; Bosch et al., 2006; Jeong et al., 2009). In the present study, the comorbidities of chronic kidney disease, diabetes, and COPD increased the risk of pneumonia infection in patients with SLE. Diabetes itself has been proven to be a major risk factor in pnemonia (for example, coronavirus disease) infection (Abdi et al., 2020). In terms of COPD, the incidence of lower respiratory tract infections was higher in patients with exacerbated COPD (Sethi, 2010). The use of corticosteroids and hydroxychloroquine also significantly increased the risk of pnemonia infection in patients with SLE, which correlated with a previous study (Kang and Park, 2003). This is mainly because immunosuppressive drugs strongly suppress immune responses against microorganisms, thereby facilitating the onset of pneumonia.

SLE affects females more frequently than males, with a ratio of nearly 9:1 (Tsang and Bultink, 2021). SLE is most commonly diagnosed during the reproductive age, which may be owing to endogenous estrogen production, failure in X chromosome inactivation, increased expression of Toll-like receptors, and alterations in microRNA function (Nusbaum et al., 2020; Leosuthamas et al., 2023). Men with SLE have a more aggressive clinical course, which include cardiovascular disease and nephritis. However, musculoskeletal involvement appears to be more common in women patients (Andrade et al., 2007; Crosslin and Wiginton, 2011). In the present study, female patients with SLE who used TCM for >60 days had a 35% reduced risk of pneumonia infection. SLE usually occurs in the women of reproductive age (Harden and Hammad, 2020); therefore, TCM use for >60 days could significantly reduce the risk of pneumonia infection in younger patients with SLE (age <65 years).

The formulae of the KF group have been used for treating chronic diseases, COPD, bone marrow suppression, and osteoprosis (Shi et al., 2014; He et al., 2017; You et al., 2019; Gao et al., 2020). In the present study, the use of formulae of the KF group for >60 days significantly reduced the risk of pneumonia infection in patients with SLE. A similar result was observed with the use of the same formulae for >90 days. Lupus nephritis affects nearly half of the patients with SLE and is one of the most commom contributors of patient mortality in SLE. In addition, SLE usually occurs in female patients during adolescence and pregnancy who are predisposed toward kidney yin deficiency (for patients with deficiency syndrome) and stagnation in the blood (for patients with excess syndrome). Furthermore, female patients easily develop kidney and liver yin deficiency during and after menopause. As per TCM, these deficiencies should be treated with a long course of TCM therapy. The formulae of the BF group have been used for treating rheumatoid arthritis, psoriasis, and coronary heart disease (Qiu et al., 2005; Gong et al., 2021; Liu et al., 2021). In the present study, a 46% reduction in the risk of pneumonia infection was observed in patients with SLE who received BF formulae for <30 days (OR = 0.48; *p* ≤ 0.001). BF formulae are usually prescribed for patients with SLE who also have arthritis, facial erythema, and erythema nodosa. Furthermore, these diseases, which belong to excess syndrome, can be ameliorated in a short period of time. However, using these formulae for a long period would consume healthy Qi. Jieduquyuziyin prescription, which contains both herbs from KF and BF formulae, has been shown to ameliorate SLE in MRL/lpr mice by inhibiting the expression of the IRAK1-NF-κB and PI3K/Akt/PGC-1α signaling pathways (Ji et al., 2020; Ji et al., 2022). Therefore, the underlying mechanisms by which TCM therapies may reduce the risk of pneumonia in patients with SLE need further investigations in the future. Moreover, more randomized clinical trials using TCM and therapies and conventional treatment would be the better way to provide the robust evidence.

However, the study had several limitations, which are inherent to observational studies and registries. For instance, detailed information regarding the SLE disease activity and the laboratory results of parameters such as anti-dsDNA, C3, and C4 were lacking. Moreover, the medication doses for each traditional Chinese herbal formula could not be fully accessed in the database. This study used a set of ICD codes to capture all types of pneumonia that occurred 1 year after the diagnosis of SLE, which may have inevitably included pneumonia caused by opportunistic pathogens as well as common pathogens, thereby confounding the results. In addition, the included study population was Taiwanese, and there may be regional differences in other regions of China and other eastern countries, including Korea and Japan, where TCM is popular for treating certain chronic diseases. Finally, the lack of some individual factors, such as the smoking status, blood pressure, body mass index, patient lifestyle, and environmental factors, may have potentially led to unmeasured confounding.

## Conclusion

The present population-based cohort study revealed that the use of TCM could reduce the risk of pneumonia in patients with SLE. In particular, the use of KF formulae for >90 days and BF formulae for <30 days are recommended in the TCM treatment of SLE. Further randomized clinical trials are needed to confirm these results.

## Data Availability

The raw data supporting the conclusion of this article will be made available by the authors, without undue reservation.
