# Supplementary material for: Traditional Chinese medicine use is associated with lower risk of pneumonia in patients with systemic lupus erythematosus: a population-based retrospective cohort study
Source: Front Pharmacol. 2023 Jun 1;14:1185809. doi: 10.3389/fphar.2023.1185809 (PMC10267408; doi:10.3389/fphar.2023.1185809)
Supplement: Supplementary file 1 [file DataSheet1.docx]

**Traditional Chinese Medicine use is associated with lower risk of Pneumonia in Patients with Systemic Lupus Erythematosus:A Population-Based Retrospective Cohort Study**

**Supplementary Data:**

**
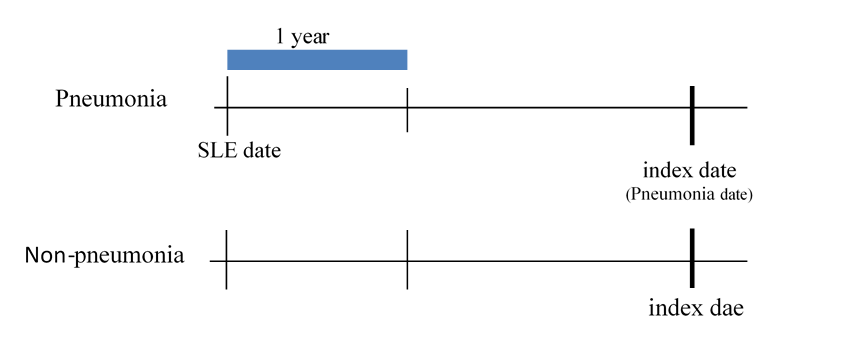
**

**Supplementary Fig. S1.** Cohort of interest, the index date.

one year of exposure time to observe the use of traditional Chinese medicine.

**Supplementary Table S1**.

The most 20 frequently prescribed Chinese herbal formulas in the NIHRD for patients (From SLE date to index date)

| No. | TCM formula | Group |
| --- | --- | --- |
| 1 | Duhuo Jisheng Decoction | BF |
| 2 | Shujin Huoxue Decoction | BF |
| 3 | Dangui Niantong Decoction | BF |
| 4 | Guilu Erxian Jiao | KF |
| 5 | Jiawei Xiaoyao Powder | Other type |
| 6 | Jisheng Shenqi Pill | KF |
| 7 | Shaoyao Gancao Decoction | BF |
| 8 | Shentong Zhuyu Decoction | BF |
| 9 | Xuefu Zhuyu Decoction | BF |
| 10 | Ma Ziren Pill | Other type |
| 11 | Qiju Dihuang Pill | KF |
| 12 | Xiangsha Liujunzi | Other type |
| 13 | Zhibai Dihuang Pill | KF |
| 14 | Gegen Decoction | Other type |
| 15 | Xiao Huoluo Dan | BF |
| 16 | Banxia Xiexin Decoction | Other type |
| 17 | Tianwang Buxin Dan | Other type |
| 18 | Guizhi Shaoyao Zhimu Decoction | BF |
| 19 | Liuwei Dihuang Pill | KF |
| 20 | Zuogui Pill | KF |

**Supplementary Table S2**. Conditional logistic regression of risk of pneumonia by age,gender,follow-up duration stratification(60days)

|  | N | No of pneumonia | aOR (95% C.I.) | p-value |
| --- | --- | --- | --- | --- |
| **Age <65^a^** |  |  |  |  |
| TCM (day) |  |  |  |  |
| None | 392 | 209 | Reference |  |
| ≤60 | 228 | 112 | 0.77 (0.53-1.12) | 0.167 |
| >60 | 206 | 92 | 0.66 (0.44-0.99) | 0.042 |
| **Age ≥ 65^a^** |  |  |  |  |
| TCM (day) |  |  |  |  |
| None | 115 | 61 | Reference |  |
| ≤60 | 73 | 40 | 1.04 (0.52-2.08) | 0.920 |
| >60 | 50 | 18 | 0.52 (0.25-1.05) | 0.069 |
| **Female^a^** |  |  |  |  |
| TCM (day) |  |  |  |  |
| None | 408 | 220 | Reference |  |
| ≤60 | 264 | 134 | 0.85 (0.60-1.20) | 0.344 |
| >60 | 232 | 98 | 0.65 (0.45-0.93) | 0.019 |
| **Male^a^** |  |  |  |  |
| TCM (day) |  |  |  |  |
| None | 99 | 50 | Reference |  |
| ≤60 | 37 | 18 | 0.58 (0.21-1.56) | 0.277 |
| >60 | 24 | 12 | 0.59 (0.19-1.77) | 0.343 |
| **Follow-up duration ≤2 years (SLE date to index date)^b^** | | | | |
| TCM (day) |  |  |  |  |
| None | 107 | 55 | Reference |  |
| ≤60 | 30 | 13 | 0.60 (0.19-1.91) | 0.387 |
| >60 | 8 | 3 | 0.28 (0.03-2.31) | 0.238 |
| **Follow-up duration >2 years (SLE date to index date)^a^** | | | | |
| TCM (day) |  |  |  |  |
| None | 400 | 215 | Reference |  |
| ≤60 | 271 | 139 | 0.84 (0.60-1.19) | 0.326 |
| >60 | 248 | 107 | 0.66 (0.46-0.93) | 0.019 |
| **Follow-up duration ≤3 years (SLE date to index date)^a^** | | | | |
| TCM (day) |  |  |  |  |
| None | 165 | 88 | Reference |  |
| ≤60 | 49 | 21 | 0.37 (0.15-0.93) | 0.034 |
| >60 | 23 | 9 | 0.48 (0.15-1.55) | 0.220 |
| **Follow-up duration >3 years (SLE date to index date)^a^** | | | | |
| TCM (day) |  |  |  |  |
| None | 342 | 182 | Reference |  |
| ≤30 | 252 | 131 | 0.90 (0.62-1.30) | 0.569 |
| >30 | 233 | 101 | 0.64 (0.44-0.93) | 0.019 |
| **Follow-up duration ≤7 years (SLE date to index date)^a^** | | | | |
| TCM (day) |  |  |  |  |
| None | 344 | 179 | Reference |  |
| ≤60 | 146 | 72 | 0.81 (0.52-1.27) | 0.361 |
| >60 | 87 | 39 | 0.66 (0.39-1.11) | 0.119 |
| **Follow-up duration >7 years (SLE date to index date)^a^** | | | | |
| TCM (day) |  |  |  |  |
| None | 163 | 91 | Reference |  |
| ≤60 | 155 | 80 | 0.77 (0.46-1.30) | 0.331 |
| >60 | 169 | 71 | 0.57 (0.35-0.94) | 0.028 |
| **Follow-up duration ≤8 years (SLE date to index date)^a^** | | | | |
| TCM (day) |  |  |  |  |
| None | 373 | 194 | Reference |  |
| ≤60 | 161 | 79 | 0.83 (0.55-1.26) | 0.383 |
| >60 | 105 | 48 | 0.67 (0.42-1.08) | 0.103 |
| **Follow-up duration >8 years (SLE date to index date)^a^** | | | | |
| TCM (day) |  |  |  |  |
| None | 134 | 76 | Reference |  |
| ≤60 | 140 | 73 | 0.85 (0.48-1.53) | 0.596 |
| >60 | 151 | 62 | 0.55 (0.32-0.95) | 0.032 |

a: Adjusted for hypertension, hyperlipidemia, chronic liver disease, chronic kidney disease, diabetes, chronic obstructive pulmonary disease, rheumatoid arthritis, ankylosing spondylitis, corticosteroids, NSAIDs, hydroxychloroquine, and methotrexate.

b: Adjusted for hypertension, hyperlipidemia, chronic liver disease, chronic kidney disease, rheumatoid arthritis, corticosteroids, NSAIDs, and hydroxychloroquine.

**Supplementary Table S3**. Sensitivity analysis of risk of pneumonia

|  | Non-Pneumonia | Pneumonia | cOR (95% C.I.) | p-value | aOR† (95% C.I.) | p-value |
| --- | --- | --- | --- | --- | --- | --- |
| **Pneumonia ER vs Non-pneumonia** | | |  |  |  |  |
| TCM (day) |  |  |  |  |  |  |
| None | 237 | 122 | Reference |  | Reference |  |
| ≤60 | 149 | 81 | 1.06 (0.75-1.50) | 0.759 | 0.97 (0.67-1.39) | 0.859 |
| >60 | 146 | 64 | 0.85 (0.59-1.23) | 0.390 | 0.79 (0.54-1.17) | 0.241 |
| **Pneumonia admission <8 days vs Non-pneumonia** | | | |  |  |  |
| TCM (day) |  |  |  |  |  |  |
| None | 237 | 87 | Reference |  | Reference |  |
| ≤60 | 149 | 46 | 0.84 (0.56-1.27) | 0.410 | 0.86 (0.55-1.34) | 0.506 |
| >60 | 146 | 27 | 0.50 (0.31-0.81) | 0.005 | 0.39 (0.23-0.66) | <0.001 |
| **Pneumonia admission ≥8 days vs Non-pneumonia** | | | |  |  |  |
| TCM (day) |  |  |  |  |  |  |
| None | 237 | 61 | Reference |  | Reference |  |
| ≤60 | 149 | 25 | 0.65 (0.39-1.08) | 0.099 | 0.70 (0.41-1.20) | 0.195 |
| >60 | 146 | 19 | 0.51 (0.29-0.88) | 0.016 | 0.46 (0.25-0.83) | 0.010 |
| **Pneumonia with use of antibiotics vs Non-pneumonia** | | | |  |  |  |
| TCM (day) |  |  |  |  |  |  |
| None | 237 | 251 | Reference |  | Reference |  |
| ≤60 | 149 | 139 | 0.88 (0.66-1.18) | 0.394 | 0.86 (0.63-1.17) | 0.332 |
| >60 | 146 | 98 | 0.63 (0.46-0.87) | 0.004 | 0.60 (0.43-0.84) | 0.003 |
| **Pneumonia admission <7 days vs Non-pneumonia** | | | |  |  |  |
| TCM (day) |  |  |  |  |  |  |
| None | 237 | 74 | Reference |  | Reference |  |
| ≤60 | 149 | 35 | 0.75 (0.48-1.18) | 0.216 | 0.74 (0.46-1.20) | 0.219 |
| >60 | 146 | 25 | 0.55 (0.33-0.90) | 0.018 | 0.43 (0.25-0.74) | 0.002 |
| **Pneumonia admission ≥7 days vs Non-pneumonia** | | | |  |  |  |
| TCM (day) |  |  |  |  |  |  |
| None | 237 | 74 | Reference |  | Reference |  |
| ≤60 | 149 | 36 | 0.77 (0.49-1.21) | 0.262 | 0.83 (0.52-1.35) | 0.459 |
| >60 | 146 | 21 | 0.46 (0.27-0.78) | 0.004 | 0.42 (0.23-0.74) | 0.003 |

†Adjusted for hypertension, hyperlipidemia, chronic liver disease, chronic kidney disease, diabetes, chronic obstructive pulmonary disease, rheumatoid arthritis, ankylosing spondylitis, corticosteroids, NSAIDs, hydroxychloroquine, and methotrexate.

**Supplementary Table S4** Conditional logistic regression of risk of pneumonia by different formulae of TCM.

|  | N | No of pneumonia | aOR† (95% C.I.) | p-value |
| --- | --- | --- | --- | --- |
| KF |  |  |  |  |
| No | 797 | 414 | Reference |  |
| Yes | 267 | 118 | 0.87 (0.60-1.27) | 0.477 |
| BF |  |  |  |  |
| No | 708 | 376 | Reference |  |
| Yes | 356 | 156 | 0.68 (0.48-0.96) | 0.030 |
| KF (days) |  |  |  |  |
| None | 797 | 414 | Reference |  |
| ≤30 | 136 | 68 | 1.26 (0.79-2.00) | 0.328 |
| 31-60 | 49 | 20 | 0.71 (0.34-1.50) | 0.368 |
| >60 | 82 | 30 | 0.41 (0.22-0.74) | 0.003 |
| BF (days) |  |  |  |  |
| None | 708 | 376 | Reference |  |
| ≤30 | 182 | 68 | 0.48 (0.31-0.73) | <0.001 |
| 31-60 | 58 | 32 | 1.14 (0.59-2.21) | 0.705 |
| >60 | 116 | 56 | 1.01 (0.61-1.68) | 0.973 |
| KF (days) |  |  |  |  |
| None | 797 | 414 | Reference |  |
| ≤90 | 203 | 93 | 0.98 (0.65-1.47) | 0.915 |
| >90 | 64 | 25 | 0.50 (0.26-0.96) | 0.038 |
| BF (days) |  |  |  |  |
| None | 708 | 376 | Reference |  |
| ≤90 | 271 | 116 | 0.64 (0.44-0.92) | 0.017 |
| >90 | 85 | 40 | 0.96 (0.54-1.72) | 0.896 |
| KF (days) |  |  |  |  |
| None | 797 | 414 | Reference |  |
| ≤30 | 136 | 68 | 1.27 (0.80-2.02) | 0.310 |
| >30 | 131 | 50 | 0.49 (0.29-0.82) | 0.007 |
| BF (days) |  |  |  |  |
| None | 708 | 376 | Reference |  |
| ≤30 | 182 | 68 | 0.48 (0.31-0.74) | <0.001 |
| >30 | 174 | 88 | 1.03 (0.66-1.61) | 0.896 |
| KF (days) |  |  |  |  |
| None | 797 | 414 | Reference |  |
| ≤60 | 185 | 88 | 1.10 (0.73-1.67) | 0.645 |
| >60 | 82 | 30 | 0.45 (0.25-0.82) | 0.008 |
| BF (days) |  |  |  |  |
| None | 708 | 376 | Reference |  |
| ≤60 | 240 | 100 | 0.59 (0.40-0.88) | 0.009 |
| >60 | 116 | 56 | 0.95 (0.57-1.57) | 0.833 |

†Adjusted for hypertension, hyperlipidemia, chronic liver disease, chronic kidney disease, diabetes, chronic obstructive pulmonary disease, rheumatoid arthritis, ankylosing spondylitis, corticosteroids, NSAIDs, hydroxychloroquine, and methotrexate.

KF: Chinese formulae tonifying the kidney; BF: Chinese formulae activating blood circulation.

**Supplemental table S5** Top 20 distribution of combined diseases with pneumonia

| Disease | N |
| --- | --- |
| 518 Other diseases of lung | 42 |
| 518.4 Acute edema of lung, unspecified | 4 |
| 518.81 Acute respiratory failure | 34 |
| 518.82 Other pulmonary insufficiency, not elsewhere classified | 4 |
| 780 General symptoms | 42 |
| 780.4 Dizziness and giddiness | 5 |
| 780.6 Fever and other physiologic disturbances of temperature regulation | 37 |
| 038 Streptococcal septicemia | 38 |
| 038.42 Escherichia coli | 3 |
| 038.9 Unspecified septicemia | 35 |
| R50 Fever of other and unknown origin | 29 |
| 710 Diffuse diseases of connective tissue | 25 |
| 599 Other disorders of urethra and urinary tract | 24 |
| 786 Symptoms involving respiratory system and other chest symptoms | 17 |
| 511 Pleurisy | 15 |
| 583 Nephritis and nephropathy, not specified as acute or chronic | 15 |
| 401 Essential hypertension | 14 |
| 428 Heart failure | 14 |
| 250 Diabetes mellitus | 13 |
| 714 Rheumatoid arthritis and other inflammatory polyarthropathies | 13 |
| 785 Symptoms involving cardiovascular system | 13 |
| 276 Disorders of fluid, electrolyte and acid-base balance | 11 |
| N18 Chronic kidney disease | 11 |
| N39 Other disorders of urinary system | 11 |
| 493 Asthma | 10 |
| 584 Acute renal failure | 10 |
| E87 Other disorders of fluid, electrolyte and acid-base balance | 10 |
